# The secretory small GTPase Rab27B as a marker for breast cancer progression

**DOI:** 10.18632/oncotarget.140

**Published:** 2010-08-05

**Authors:** An Hendrix, Geert Braems, Marc Bracke, Miguel C. Seabra, William A. Gahl, Olivier De Wever, Wendy Westbroek

**Affiliations:** ^1^Department of Medical Oncology, Ghent University Hospital, De Pintelaan 185, 9000 Ghent, Belgium; ^2^Department of Gynecology, Ghent University Hospital, De Pintelaan 185, 9000 Ghent, Belgium; ^3^Laboratory of Experimental Cancer Research, Department of Radiation Oncology and Experimental Cancer Research, Ghent University Hospital, De Pintelaan 185, 9000 Ghent, Belgium; ^4^Molecular Medicine, National Heart and Lung Institute, Imperial College London, Sir Alexander Fleming Building, Exhibition Road, London SW7 2AZ, UK; ^5^CEDOC, Faculdade de Ciências Médicas, Universidade Nova de Lisboa, Lisbon, Portugal; ^6^Instituto Gulbenkian de Ciência, Oeiras, Portugal; ^7^Medical Genetics Branch, National Human Genome Research Institute, 10 Center Drive, Bethesda, MD 20892, USA

## Abstract

In contemporary oncology practice, an urgent need remains to refine the prognostic assessment of breast cancer. It is still difficult to identify patients with early breast cancer who are likely to benefit from adjuvant chemotherapy. Although invasion of cancer cells is the main prognostic denominator in tumor malignancy, our molecular understanding and diagnosis are often inadequate to cope with this activity. Therefore, deciphering molecular pathways of how tumors invade and metastasize may help in the identification of a useful prognostic marker. We recently discovered that the secretory small GTPase Rab27B, a regulator of vesicle exocytosis, delivers proinvasive signals for increased invasiveness, tumor size, and metastasis of various estrogen receptor (ER)-positive breast cancer cell lines, both in vitro and in vivo. In human breast cancer specimens, the presence of Rab27B protein proved to be associated with a low degree of differentiation and the presence of lymph node metastasis in ER-positive breast cancer.

## INTRODUCTION

Breast cancer remains a top-priority in health care. The world-wide number of new cases was estimated 1,15 million for 2002, only surpassed by lung cancer when taking both sexes together. Due to the relatively positive prognosis there were 4,4 million survivors up to 5 years after diagnosis world-wide. Nevertheless 411 000 annual deaths were reported, being the leading cause of cancer mortality in women [1]. Estrogen receptor (ER)-positive breast cancers, which comprise the majority of breast malignancies, carry a better prognosis for disease-free survival and overall survival than ER-negative breast cancers. However, a significant percentage of women with ER-positive breast cancer will die from the disease within 10 years [2]. Furthermore, time-dependent tumor characteristics currently used in the clinic such as tumor size and lymph node status are generally less developed in early breast cancer and may be less declarative of risk. These examples indicate that more accurate prognostic indicators would help in the selection of patients at high risk for disease recurrence and death who are candidates for systemic adjuvant therapy. Deciphering molecular mechanisms of cancer cell invasion, the main prognostic denominator in tumor malignancy, may identify useful prognostic markers to cope with this need. We investigated a novel pro-invasive pathway, namely the delivery of critical factors by Rab GTPases into the tumor ecosystem [3].

## RAB27B-MEDIATED VESICLE TRANSPORT REGULATES BREAST CANCER CELL GROWTH AND INVASION

Vesicular transport is the basic communication mechanism between different compartments within a cell as well as with the extracellular environment, and consists of two membrane trafficking networks: endocytosis and exocytosis. Both systems directly impact cell signaling: endocytosis regulates the internalization of receptors and thereby modulates responses to external stimuli, and exocytosis affects signaling by liberating vesicles and signaling molecules [4]. Rab GTPases are intracellular transport proteins that master vesicle trafficking. The activity of the small GTPases is regulated by changes in guanine nucleotide binding status. Activated Rabs allow vesicles to engage specific effectors required for vesicle movement, docking and fusion [5]. Secretory Rab GTPases control regulated vesicle exocytosis [6]. After cloning the secretory *Rab27A* and *Rab27B* genes [7], our group has continued to study the cellular function of this Rab subfamily [3, 8-10]. The secretory Rab27 subfamily consists of the homologues Rab27A and B; they exhibit 71% identity at the amino acid level. Recently, we demonstrated that Rab27B regulates invasive tumor growth and metastasis of ER-positive MCF-7, T47D and ZR75.1 breast cancer cells using complementary cell culture and xenograft mouse models [3]. Overexpression of the Rab27B protein to comparable expression levels found in poor prognosis primary breast cancer resulted in G1 to S phase cell cycle transition, growth and invasiveness of cells in cell culture, and invasive tumor growth and hemorrhagic ascites production in nude mice. Interestingly, we found no such effects with Rab27A in vitro and in vivo [3]. Common, but also different non- redundant functions of Rab27A versus Rab27B have been described dependent on the cell type and/or secretory process studied [11-14]. Interestingly, Wang et al. (2008) reported Rab27A-dependent invasion and xenograft metastasis of ER-negative, already invasive, MDA- MB-231 and MDA-MB-435 breast cancer cells. Rab27A mRNA and protein levels increased with the in vitro invasive potential of the breast cancer cells studied [15].

**Figure 1:**
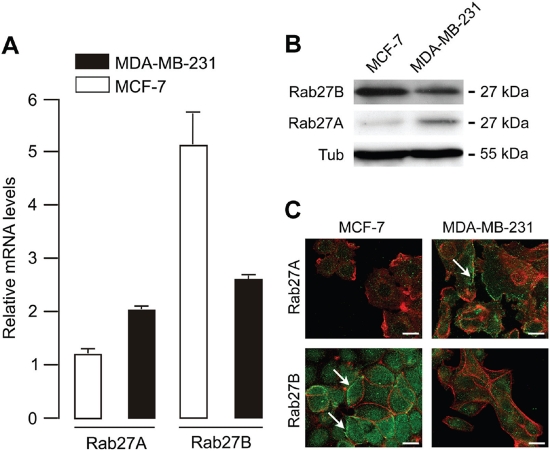
Rab27A and Rab27B mRNA and protein expression in MCF-7 and MDA-MB-231 breast cancer cells. (A) Rab27A and Rab27B mRNA expression detected via quantitative real-time PCR (relative to human mammary epithelial cell line MCF10A). To demonstrate Rab27A and Rab27B mRNA expression we combined 50 ng cDNA, Taqman gene expression master mix reagent and Assays-On- Demand (Applied Biosystems, Austin, TX) for *Rab27B* (Assay ID Hs00188156_m1), *Rab27A* (Assay ID Hs00608302_m1), and the household gene *PIAA* (Assay ID Hs99999904_m1) on an ABI PRISM 7900 HT Sequence Detection System (Applied Biosystems) using the 2^−ΔΔ^CT method for relative gene expression. The cycling conditions comprised 2 min at 50°C, 10 min at 95°C and 40 cycles at 95°C for 15 s and 60°C for 60 s. (B) Rab27A and Rab27B protein expression detected via Western blot analysis using the specific goat polyclonal Rab27A antibody (C-20, Santa Cruz Biotechnology) and our specific rabbit polyclonal Rab27B antibody. (C) Laser scanning confocal images showing endogenous localization of Rab27A and Rab27B (green) and F-actin (red) in MDA-MB-231 and MCF-7 breast cancer cells. Scale bar: 20 μm.

**Figure 2:**
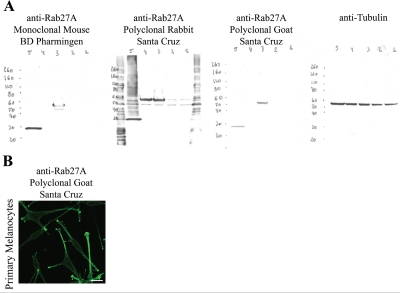
Rab27A antibody testing for Western blot analysis and laser scanning confocal microscopy. (A) Different Rab27A antibodies were tested for Western blot analysis using HEK cells transiently transfected with (1) GFP, (2) GFP-Ra-b3A, (3) GFP-Rab27A or (4) GFP-Rab27B and (5) are primary melanocytes. Tubulin was used as loading control. (B) Laser scanning confocal microscopy of primary melanocytes with the specific goat polyclonal Rab27A antibody (C-20, Santa Cruz). Scale bar: 20 µm.

Using semi-quantitative real-time RT-PCR and primers and PCR conditions that were not specified, Wang et al. found no Rab27B mRNA expression in non-invasive, ER-positive MCF-7 cells, and in invasive ER-negative MDA-MB-231 cells [15]. In contrast, we observed both Rab27A and Rab27B expression in these cells, employing quantitative real-time PCR and Western blotting (Fig. 1A and B). In fact, the expression levels of Rab27B mRNA and protein were higher in ER-positive MCF-7 cells compared to ER-negative MDA-MB-231 cells; Rab27A mRNA and protein levels were higher in ER-negative MDA-MB-231 cells. In the study of Wang et al. Rab27A was found diffusely localized in the cytoplasm, with a particular concentration in the perinuclear region of MDA-MB-231 and MDA-MB-435 cells. Using laser scanning confocal microscopy we revealed a vesicular distribution for both Rab27A and Rab27B in MCF-7 and MDA-MB-231 cells (Fig. 1C). Rab27B exhibits a peripheral distribution in MCF-7 cells and a cytoplasmic pattern in MDA-MB-231 cells; Rab27A is localized at the cell periphery in MDA-MB-231 cells and in the cytoplasm in MCF-7 cells. Both we and Wang et al. obtained the cell lines directly from ATCC [3, 15]. Differences in Rab27 expression and localization could be attributed to failure of Rab27B-specific primers in the semi-quantitative PCR assay or the lack of specificity of the Rab27A antibody used by Wang and coworkers. For Western blot analysis, we tested three distinct Rab27A antibodies on the cell lysates of HEK cells electroporated with previously described GFP-Rab27A, GFP-Rab27B or GFP-Rab3A constructs [3, 8] (Fig. 2A); One of the Rab27A antibodies was non-specific (rabbit polyclonal antibody, Santa Cruz Biotechnology). Results in Fig. 1B were obtained using the specific goat polyclonal Rab27A antibody (C-20, Santa Cruz Biotechnology). Specificity of the rabbit polyclonal Rab27B antibody has been shown previously [3, 11]. Evaluation of the specific goat polyclonal Rab27A antibody for laser scanning confocal microscopy was performed on primary human melanocytes, a cell type with a well studied expression and localization pattern of Rab27A [10, 16] (Fig. 2B).

The differences in localization and expression of both Rab27 proteins in ER-positive and negative breast cancer cell lines could indicate that Rab27A and Rab27B play different roles depending on the cells’ ER-status. Overexpression of Rab27B in non-invasive ER-positive breast cancer cells comparable to levels found in poor-prognosis primary breast tumors, stimulated invasive tumor growth. Rab27B localized to the cell periphery, suggesting a secretory function. Since estrogen regulates vesicle trafficking gene expression in ER-positive breast cancer cells [17], it is of interest to determine Rab27A and Rab27B mRNA and protein levels in additional estrogen-dependent carcinomas such as ovarian carcinoma. Also, conflicting reports in the literature suggest that Rab25, involved in the recycling of cell surface receptors and integrins, could be a context dependent promoter or suppressor of tumorigenesis. It has been demonstrated that in ER-positive breast cancer cells, Rab25 overexpression increases proliferation and anchorage-independent growth [18]. In ER-negative breast cancer cells, re-expression of Rab25 reduced proliferation and invasive growth, and increased apoptosis [19, 20].

## RAB27B: A PROGNOSTIC MARKER FOR ER-POSITIVE BREAST CANCER?

Our previous findings indicate that in clinical breast cancer samples, up-regulation of endogenous Rab27B, but not Rab27A, protein correlates with lymph node metastasis and differentiation grade in ER-positive breast tumors [3]. In agreement, levels of Rab27B mRNA were highest in ER-positive breast cancers with lymph node metastasis. Based on this body of evidence, Rab27B determines invasiveness and metastasis, and could be an important biomarker in the signature of ER-positive breast cancers with poor prognosis. The selective presence of Rab27B expression in ER-positive tumors might suggest Rab27B expression as a confounding factor. By definition, a confounding variable is associated with both cause and outcome. However, no significant relationship was found for the percentage of ER-positivity and lymph node status or possible confounder Rab27B. Moreover, using percentage ER-positivity, grading, diameter of the tumor, and Rab27B score as variables in a logistic regression model, only Rab27B score was significantly linked to lymph node metastasis (Odds Ratio = 10.1; 95% Confidence Interval = 1.67 to 60.93; *P* = 0.01) (Clinical data are available in Supplementary Table 2 in [3]).

## CONCLUSION

Breast cancer is a heterogeneous disease and its biological complexity is a major challenge for translational research. In the clinic, additional well-defined biological markers could improve breast cancer sub-classification and the accuracy of prognostic and therapeutic decisions for a tailormade therapy. Use of markers with an affordable and widely spread technology, such as immunohistochemistry, is of advantage. Rab27B is a potential key marker for stratification, prognosis and treatment of early stage ER-positive breast cancers which are more invasive and tend to metastasize more frequently.
